# Health-Related Quality of Life of Young Adults Treated with Recombinant Human Growth Hormone during Childhood

**DOI:** 10.1371/journal.pone.0140944

**Published:** 2015-10-16

**Authors:** Grit Sommer, Micol E. Gianinazzi, Rahel Kuonen, Julia Bohlius, Dagmar l’Allemand, Michael Hauschild, Primus-Eugen Mullis, Claudia E. Kuehni, Swiss Society for Paediatric Endocrinology and Diabetology (SGPED)

**Affiliations:** 1 Institute of Social and Preventive Medicine, University of Bern, Bern, Switzerland; 2 Clinic of Adolescent Medicine, Division of Paediatric Endocrinology and Diabetology, Ostschweizer Kinderspital, St. Gallen, Switzerland; 3 Division of Paediatric Endocrinology, Diabetology and Obesity, Department of Paediatrics, University Hospital, Lausanne, Switzerland; 4 Division of Paediatric Endocrinology, Diabetology & Metabolism, University Children’s Hospital Bern, Inselspital, Bern, Switzerland; Xi'an Jiaotong University School of Medicine, CHINA

## Abstract

**Background:**

Since recombinant human growth hormone (rhGH) became available in 1985, the spectrum of indications has broadened and the number of treated patients increased. However, long-term health-related quality of life (HRQoL) after childhood rhGH treatment has rarely been documented. We assessed HRQoL and its determinants in young adults treated with rhGH during childhood.

**Methodology/Principal Findings:**

For this study, we retrospectively identified former rhGH patients in 11 centers of paediatric endocrinology, including university hospitals and private practices. We sent a questionnaire to all patients treated with rhGH for any diagnosis, who were older than 18 years, and who resided in Switzerland at time of the survey. Three hundred participants (58% of 514 eligible) returned the questionnaire. Mean age was 23 years; 56% were women; 43% had isolated growth hormone deficiency, or idiopathic short stature; 43% had associated diseases or syndromes, and 14% had growth hormone deficiency after childhood cancer. Swiss siblings of childhood cancer survivors and the German norm population served as comparison groups. HRQoL was assessed using the Short Form-36. We found that the Physical Component Summary of healthy patients with isolated growth hormone deficiency or idiopathic short stature resembled that of the control group (53.8 vs. 54.9). Patients with associated diseases or syndromes scored slightly lower (52.5), and former cancer patients scored lowest (42.6). The Mental Component Summary was similar for all groups. Lower Physical Component Summary was associated with lower educational level (coeff. -1.9). Final height was not associated with HRQoL.

**Conclusions/Significance:**

In conclusion, HRQoL after treatment with rhGH in childhood depended mainly on the underlying indication for rhGH treatment. Patients with isolated growth hormone deficiency/idiopathic short stature or patients with associated diseases or syndromes had HRQoL comparable to peers. Patients with growth hormone deficiency after childhood cancer were at high risk for lower HRQoL. This reflects the general impaired health of this vulnerable group, which needs long-term follow-up.

## Introduction

When recombinant human growth hormone (rhGH) was introduced in 1985, it was only approved for short children with growth hormone deficiency (GHD). Today, approved indications for rhGH treatment include GHD after cancer treatment, Turner syndrome, chronic renal failure, Prader-Willi-Syndrome, born small for gestational age (SGA), short stature homeobox deficiency, and, in some countries, idiopathic short stature (ISS). As the number of indications has grown, so has the number of children treated with rhGH. Most frequently, rhGH treatments are intended to promote growth, but rhGH therapy also improves body composition, cardiovascular outcomes, lipid profile, and bone density [1].

In adult patients, rhGH treatment improves health-related quality of life (HRQoL) [2, 3]. But it is not clear if treating patients with rhGH during childhood improves their long-term HRQoL, because study results are contradictory [4]. Their results are hard to compare because the studies are designed differently (time of assessment, use of different HRQoL instruments, patient samples and control groups) [4]. Most research groups assessed HRQoL after patients had initiated rhGH treatment, and few addressed long-term HRQoL after treatment [5–7]. Previous studies compared GHD patients with their siblings [6], norm populations [5], or themselves (longitudinally, at different time points) [8–10]. However, it is unclear which untreated comparison group is appropriate. Many publications on HRQoL focused on single disorders, like GHD, Turner syndrome, ISS, children born SGA or survivors of childhood cancer. Others investigated heterogeneous cohorts that contained patients with different indications. It is poorly known how widely HRQoL varies between patients with different underlying indications for rhGH treatment.

The current study aims to investigate this. We assessed HRQoL in young adults who had been treated with rhGH during childhood, and to determine if the indication for rhGH treatment, or other factors, influenced long-term HRQoL. We used the standardized questionnaire Short Form 36 (SF-36) to collect data on HRQoL, and focused on long-term HRQoL in patients whose linear growth was complete. We used both Swiss healthy untreated siblings of childhood cancer survivors and the German norm population as comparison group.

## Subjects and Methods

This study is based on data from the Swiss Growth Registry. It uses clinical data from original medical charts and patient-reported answers to a postal questionnaire. The questionnaire was sent in the framework of a large European study on “Safety and Appropriateness of Growth hormone treatments in Europe” (SAGhE) (http://cordis.europa.eu/result/rcn/57069_en.html). This paper reports national data from Switzerland only.

### Population and study design

For this cohort study, we retrospectively identified patients treated with rhGH since 1985 in 11 different Swiss centres of paediatric endocrinology. We included patients who were alive, born before 31 March 1993 (aged ≥18 years by the time of the study), and resident in Switzerland. These patients had been treated with rhGH for the following indications: isolated GH Deficiency (IGHD); Turner syndrome; cancer; multiple pituitary hormone deficiency (MPHD); children born SGA; ISS; or, GHD associated with other defined diagnoses. We excluded patients with unclear diagnoses or chronic renal failure.

In 2011, eligible participants received an information letter about the study and a questionnaire with a prepaid return envelope from their former paediatric endocrinology clinic. Non-responders were mailed a reminder letter that included another copy of the questionnaire 4–6 weeks later. If they still did not reply, we reminded them by phone, or, if no phone number was available, we sent them a third reminder letter.

### Control group

We used two different comparison groups: Swiss healthy untreated controls and the German norm population. We had recruited Swiss controls in a previous study, where we asked survivors of childhood cancer for consent to contact their siblings [11–13]. In 2010 and 2011, these siblings received a questionnaire that contained the SF-36. The questionnaire was comparable to that sent to rhGH patients, but did not have questions about their history of rhGH treatment. The siblings only received one reminder letter. We also used norm data from the German Federal Health Survey, from which we selected a subgroup similar to our cohort in age and sex distribution [14].

### Ethics approval

We received ethics approval, through the Swiss Growth Registry, from the Swiss Federal Commission of Experts for Professional Secrecy in Medical Research, and through *non-obstat* statements from the ethics committees of the cantons of Bern and Zurich. In 2014, legislation on research involving humans in Switzerland changed. We have renewed ethics approval at the cantonal ethics commission of Bern.

### Explanatory variables extracted from medical files

We extracted baseline demographic data and medical information on diagnosis and treatment from original medical files kept by participating Swiss centres of paediatric endocrinology. The data included age, sex, underlying indication for rhGH treatment, rhGH dose, age at start and at end of rhGH treatment, height at start and at end of rhGH treatment, and final height (for definition see end of paragraph). We classified diagnoses according to the European Society for Paediatric Endocrinology system [15]. Patients who received rhGH after treatment of childhood cancer were classified according to the International Classification of Childhood Cancer [16]. We stratified patients into three groups, based on their indication for rhGH treatment: 1) Group I, healthy rhGH treated patients with IGHD and ISS; 2) Group II, patients with associated diseases or syndromes; and, 3) Group III, childhood cancer survivors (S1 Table). Where data on rhGH dose was missing, we used the mean dose, between the previous and the next visit, to impute it. If the dose for the next visit was also missing, we carried the last dose forward until a rhGH dose was again listed. If we could not determine age at start of rhGH treatment from the medical files, we used age at the first indicated rhGH administration, which was routinely 6 weeks after treatment’s start. Missing age at the end of rhGH treatment was replaced with the age at which patients achieved final height (marked by the end of a six-month period during which patients grew 1 cm or less, or when they reached 18 (for women) or 20 (for men).

### Explanatory variables from the questionnaire

We assessed the education level of participants from the questionnaire [11, 17]. Levels ranged from primary, to secondary, to tertiary, as well as unknown.

### Assessment of health-related quality of life

We assessed HRQoL using the Short Form-36 (SF-36) [18], a psychometrically validated instrument that has been successfully applied in patients treated with rhGH in childhood [6, 19–22]. It consists of 36 questions that can be summarized into eight scales: physical functioning; role limitation due to physical health (role limitation physical); bodily pain; general health perception; energy and vitality; social functioning; role limitation due to emotional problems (role limitation emotional); and, mental health. The eight scales can be further aggregated into a Physical Component Summary (PCS) and a Mental Component Summary (MCS) [18]. We converted raw scores into T-scores (mean = 50, SD = 10, range 0–100) based on age- and sex-stratified norm data from a public use-file of the German Federal Survey (N = 6964) [14]. Higher scores indicate better HRQoL.

### Statistical Analyses

We compared rhGH patients who participated in the survey with these who did not, using chi square tests for categorical variables, and t-tests for continuous variables. We also compared rhGH treated participants to Swiss controls. We used appropriate weights to standardize on age and sex to ensure that the marginal distribution in the stated variables of the Swiss controls was identical to that in rhGH patients.

The means of the eight SF-36 scales and the two summary scores were used to compare the HRQoL of rhGH patients and Swiss controls. We then stratified rhGH patients by indication group and compared them to controls in a linear regression model.

In rhGH patients only, we performed univariable and multivariable linear regressions to identify factors associated with physical and mental summary scores. In the multivariable regressions, we included all variables that had been significantly associated with one of the two outcomes (p≤0.05) in the univariable analysis. We assumed the *a priori* importance of sex, therapy duration; rhGH dose received, height gain and final height, and included them in both models, whether or not they were statistically significant. We used likelihood ratio tests to compare models. Implementing several variables in a linear regression model does not interfere with the precision of its estimates [23]. We performed all analyses using Stata 12.0 (StataCorp, College Station, TX).

## Results and Discussion

### Characteristics of the Study Population

**Fig 1:**
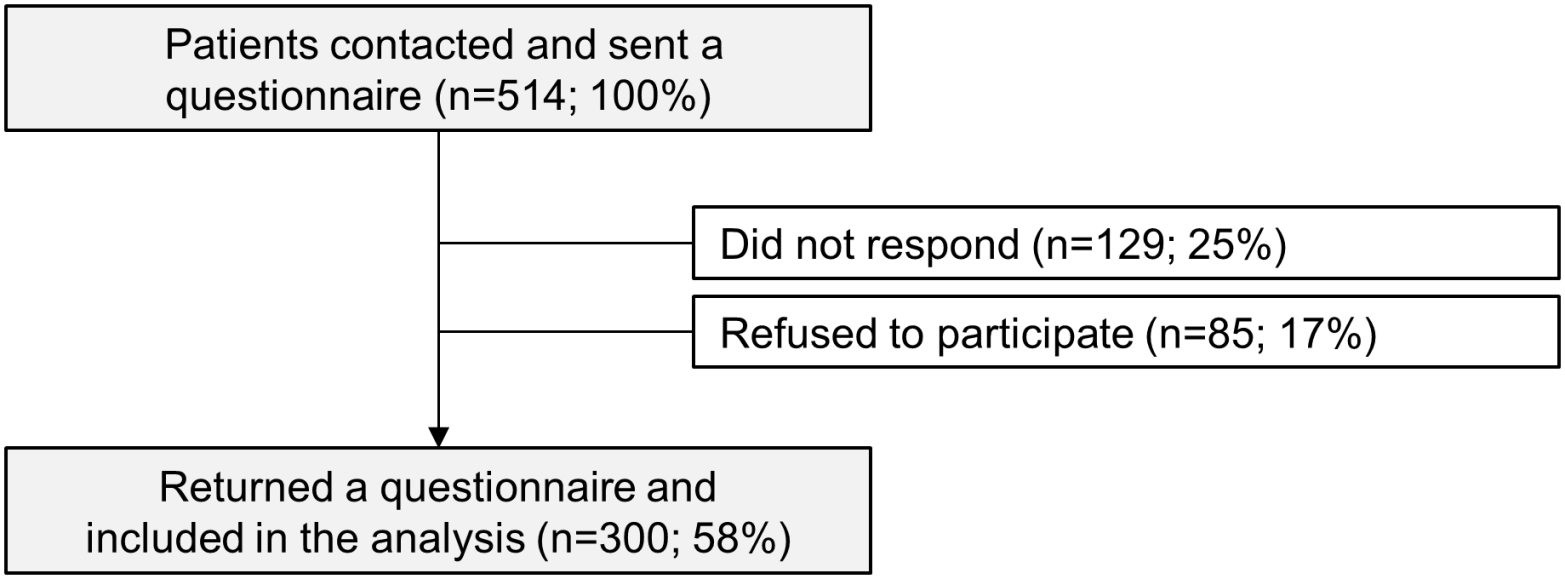
Participants and response rate of the questionnaire survey. Fig 1 shows the flow diagram of our study population starting from those who were contacted, and tapering to those included in the analysis. Patients were included if they received rhGH during childhood, were alive, ≥18 years old at time of survey, and resident in Switzerland. We excluded those with unclear diagnoses or chronic renal failure. Nine patients could not be contacted due to an unknown address. Abbreviations: rhGH, recombinant human growth hormone; SCCSS, Swiss Childhood Cancer Survivor Study.

We contacted 514 eligible rhGH patients; 300 (58%) answered the SF-36 (Fig 1). Of the 1355 Swiss controls that we contacted, 695 (51%) answered the SF-36.

Fifty-six per cent of the participants were women; their average age was 23 years. Most participants reached a secondary educational level (43%). The most common indication group for rhGH treatment was IGHD (37%), followed by Turner syndrome (16%), cancer (14%), MPHD (13%), SGA (6%), ISS (6%), and other indications (8%). Most participants (61%) had received an rhGH dose of between 30 and 50 μg/kg/day, and mean rhGH dose was 37 μg/kg/day. Mean age at start of rhGH treatment was 10 years; mean age at the end of rhGH treatment was 16 years; mean rhGH treatment duration was 6 years; mean height gain was 1.2 standard deviation score (SDS); and, mean final height was -1.0 SDS (Table 1).

**Table 1:** Characteristics of the study population: comparison of non-participants, participants and Swiss controls.

	rhGH treated non-participants^a^ (n = 214)		rhGH treated participants (n = 300)			Swiss controls^b^ (n = 695)		
	**n**	**%** ^**c**^	**n**	**%** ^**c**^	**p-value** ^**d**^	**n**	**%** ^**c**^	**p-value** ^**e**^
*Sex*					<0.001			n.a.^b^
Male	131	61	131	44		306	44	
Female	83	39	169	56		389	56	
*Current age (years)*					0.396			n.a.^b^
<20	56	26	95	32		222	32	
20–25	97	45	124	41		285	41	
>25	61	29	81	27		188	27	
*Education*	n.a.^f^	n.a.^f^						<0.001
Primary			85	28		28	4	
Secondary			129	43		297	43	
Tertiary			61	20		245	35	
Unknown			25	9		125	18	
*Indication for rhGH treatment*					<0.001	n.a.^g^	n.a.^g^	
IGHD	101	47	112	37				
ISS	3	1	17	6				
Turner	32	15	47	16				
MPHD	32	15	40	13				
SGA	14	7	18	6				
Other^h^	25	12	23	8				
Cancer	7	4	43	14				
*Indication group* ^i^					<0.001	n.a.^g^	n.a.^g^	
Group I	104	49	129	43				
Group II	103	48	128	43				
Group III	7	3	43	14				
*rhGH dose (μg/kg/day)*					0.310	n.a.^g^	n.a.^g^	
<30	45	23	75	27				
30–50	119	61	170	61				
>50	31	16	32	12				
	**mean**	**(SD)**	**mean**	**(SD)**	**p-value** ^**k**^	**mean**	**(SD)**	**p-value** ^**k**^
*Current age (years)*	23.2	3.6	22.9	4.1	0.434	n.a.^g^	n.a.^g^	
*rhGH dose (μg/kg/day)*	39.0	10.8	37.2	11.1	0.073	n.a.^g^	n.a.^g^	
*Age at start of treatment (years)*	10.5	3.0	10.2	3.3	0.195	n.a.^g^	n.a.^g^	
*Age at end of treatment (years)*	15.8	2.4	16.5	2.7	0.010	n.a.^g^	n.a.^g^	
*Treatment duration (years)*	5.2	3.0	6.3	3.8	0.001	n.a.^g^	n.a.^g^	
*Height gain (SDS)*	1.1	0.8	1.2	1.1	0.312	n.a.^g^	n.a.^g^	
*Final height (SDS)*	-0.2	0.9	-1.0	1.0	0.060	n.a.^g^	n.a.^g^	

NOTE: Percentages are based upon available data for each variable.

Abbreviations: GHD, growth hormone deficiency; IGHD, isolated growth hormone deficiency; ISS, idiopathic short stature; MPHD, multiple pituitary hormone deficiency; n, number; n.a., not applicable/ not available; rhGH, recombinant human growth hormone; SD, standard deviation; SDS, standard deviation score; SGA, small for gestational age

^a^Non-participants include 37 who were not included into mailing, 129 who did not respond and 85 who refused to participate.

^b^Age and sex standardized numbers and percentages are given for Swiss controls.

^c^Column percentages are given.

^d^p-value calculated from chi-square statistics comparing rhGH patients participants vs. non participants.

^e^p-value calculated from chi-square statistics comparing rhGH patients participants vs. Swiss controls.

^f^Information not available for non-responders.

^g^Information on rhGH treatment is not applicable for Swiss controls.

^h^Other indications include calciopenic rickets, osteogenesis imperfecta, central diabetes insipidus, clinically defined syndromes (except Turner syndrome), skeletal dysplasia, insufficient nutrient intake, disorders in organ systems, psychosocial growth failure, congenital adrenal hyperplasia.

^i^Group I includes healthy patients with IGHD or ISS; Group II patients with associated diseases or syndromes; Group III childhood cancer survivors with GHD.

^k^p- value calculated on two-sample mean-comparison test (t-test).

Participants (n = 300) differed from non-participants (n = 214) by sex, indication, age at therapy end, and therapy duration (Table 1). Both rhGH patients and Swiss controls had most commonly attained a secondary educational level.

### Health-related quality of life in rhGH patients and controls

When we compared the control groups (German norm population and Swiss controls) we found that the German norm population had lower scores on all scales, except for role limitation emotional.

Overall, the scores of patients treated with rhGH were significantly lower than those of Swiss controls in the areas of physical functioning (mean of 48.7 vs. 53.1; p<0.001), role limitation physical (49.3 vs. 50.9; p = 0.004), general health perception (52.8 vs. 56.5; p<0.001), and the PCS (51.7 vs. 54.9; p<0.001; S2 Table). We found no differences in bodily pain, energy & vitality, mental health, social functioning and role limitation emotional, and the MCS. Internal consistency was excellent for bodily pain. It was good for physical functioning, role limitation physical, energy and vitality, mental health, role limitation emotional and social functioning, and was acceptable for general health perception (Cronbach’s alpha ranging from 0.76–0.91).

**Fig 2:**
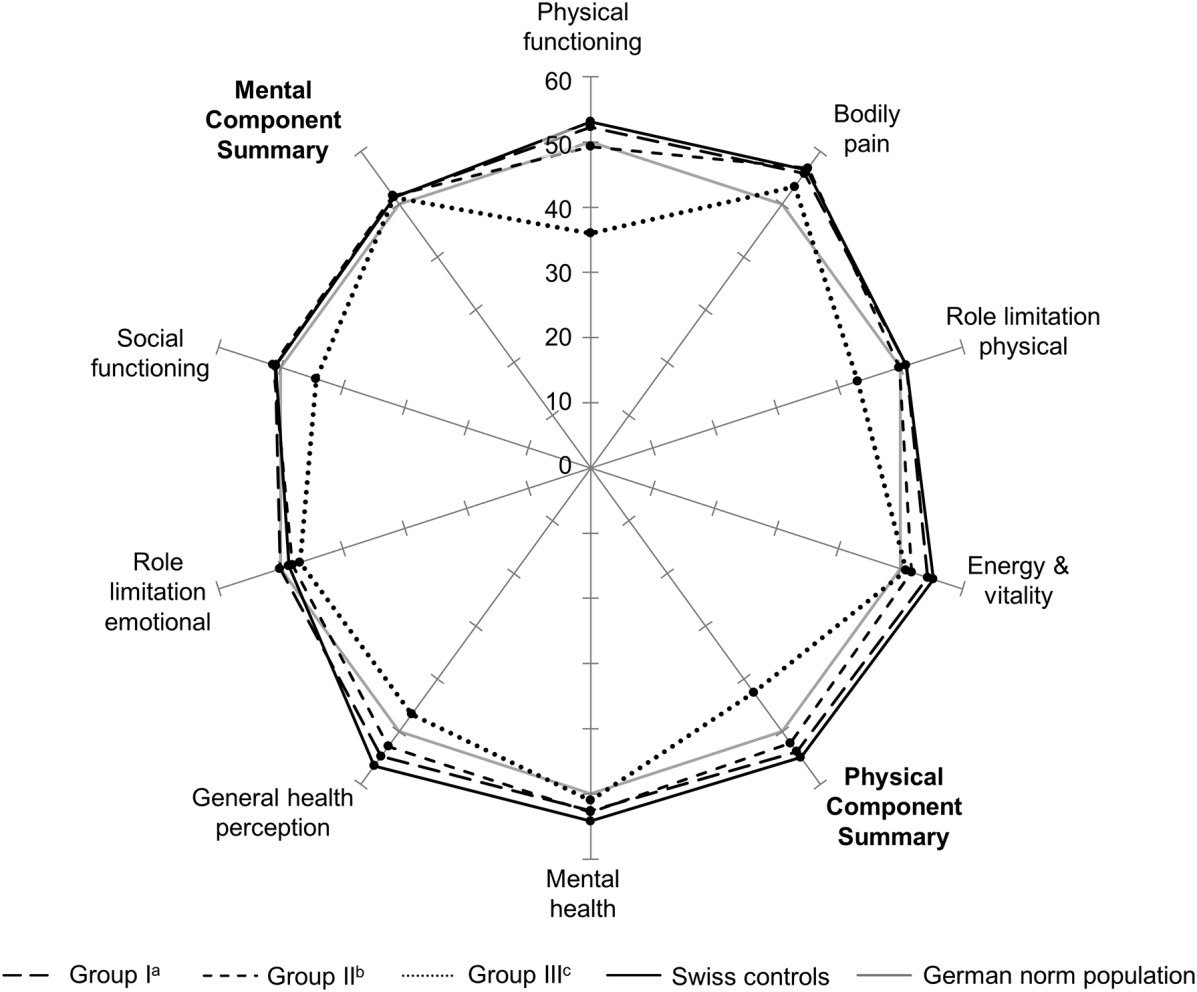
SF-36 mean T-scores of rhGH-treated patients, stratified by indication groups and compared to Swiss controls. Fig 2 shows mean T-scores for the eight SF-36 subscales and the two summary scores of rhGH treated patients, stratified by indication groups and compared to Swiss controls. Swiss controls means were unadjusted. Higher T-scores indicate higher HRQoL (expected mean from German norm population = 50, SD = 10). P-values to compare means between rhGH treated patients and Swiss controls were calculated using linear regression models.^a^Group I includes healthy patients with IGHD or ISS; ^b^Group II patients with associated diseases or syndromes; ^c^Group III childhood cancer survivors with GHD. Abbreviations: GHD, growth hormone deficiency; IGHD, isolated growth hormone deficiency; ISS, idiopathic short stature; rhGH, recombinant human growth hormone; SF-36, Short Form-36.

When we stratified the rhGH patients by indication group (Table 2 and Fig 2), we found that mean scores of patients in Groups II and III were significantly different from those of Swiss controls. Group II scored lower in the following scales: physical functioning (mean of 49.3 vs. 53.1); general health perception (52.8 vs. 56.5); and, the PCS (52.5 vs. 54.9). Patients in Group III had the lowest scores in all scales. Their scores for physical functioning (36.0 vs. 53.1), role limitation physical (43.1 vs. 50.9), general health perception (46.7 vs. 56.5), social functioning (44.3 vs. 50.8), and the PCS (42.6 vs. 54.9) were significantly lower.

**Table 2:** SF-36 mean T-scores and confidence intervals of rhGH treated patients, stratified by indication groups and compared to Swiss controls.

		Group I^a^	Group II^a^	Group III^a^	Swiss controls	p-value^b^
		(n = 129)	(n = 128)	(n = 43)	(n = 695)	
*Physical functioning*	Mean	52.3	49.3	36.0	53.1	<0.001
	95% CI	51.3, 53.3	47.3, 51.3	31.0, 41.0	52.6, 53.6	
*Bodily pain*	Mean	55.8	56.8	53.3	56.5	0.119
	95% CI	54.3, 57.2	55.4, 58.2	50.6, 56.1	55.8, 57.2	
*Role limitation physical*	Mean	50.9	49.8	43.1	50.9	<0.001
	95% CI	50.0, 51.9	48.4, 51.2	40.1, 46.0	50.3, 51.5	
*Energy & vitality*	Mean	54.4	55.5	50.9	55.3	0.164
	95% CI	51.8, 56.9	53.3, 57.6	47.2, 54.6	53.9, 56.6	
*Mental health*	Mean	52.6	52.8	51.0	54.2	0.184
	95% CI	50.4, 54.8	50.8, 54.7	47.7, 54.2	53.1, 55.3	
*General health perception*	Mean	54.7	52.8	46.7	56.5	<0.001
	95% CI	52.8, 56.6	50.8, 54.8	42.3, 51.0	55.5, 57.5	
*Role limitation emotional*	Mean	50.1	48.1	46.9	48.7	0.070
	95% CI	48.9, 51.4	46.5, 49.7	44.2, 49.6	47.8, 49.6	
*Social functioning*	Mean	50.9	51.2	44.3	50.8	0.005
	95% CI	49.3, 52.6	49.6, 52.8	40.8, 47.8	49.9, 51.6	
*PCS*	Mean	53.8	52.5	42.6	54.9	<0.001
	95% CI	52.7, 54.9	51.0, 54.1	39.0, 46.2	54.3, 55.5	
*MCS*	Mean	51.4	51.6	51.3	51.3	0.997
	95% CI	49.3, 53.6	49.6, 53.6	48.1, 54.5	50.1, 52.5	

Higher T-scores indicate higher HRQoL (expected mean from German norm population = 50, SD = 10).

Abbreviations: 95% CI, 95% confidence interval; GHD, growth hormone deficiency; IGHD, isolated growth hormone deficiency; ISS, idiopathic short stature; MCS, mental component summary; n, number; PCS, physical component summary; rhGH, recombinant human growth hormone; SF-36, Short Form-36.

^a^Group I includes healthy patients with IGHD or ISS; Group II patients with associated diseases or syndromes; Group III childhood cancer survivors with GHD.

^b^Global p-values were calculated from linear regression models, testing if the variable ‘indication group’ as a whole was associated with SF-36 mean T-scores.

To investigate differences in mean scores for single disorders within Group I and II, we split the rhGH patients by underlying indication (Table 3). The HRQoL of patients with IGHD, Turner syndrome, and those born SGA was similar to that of Swiss controls, except in the categories of physical functioning (Turner, 51.0 vs. 53.1), bodily pain (Turner, 59.0 vs. 56.5), general health perception (IGHD, 53.6 vs. 56.5), and the PCS (IGHD, 53.2 vs. 54.9). Persons with ISS (N = 17) scored higher than Swiss controls in bodily pain (59.7 vs. controls, 56.6), role limitation physical (53.3 vs. 50.9), general health perception (61.5 vs. 56.5), role limitation emotional (52.6 vs. 48.7), and the PCS (57.4 vs. 54.9). In contrast, patients with MPHD (N = 40) had lower scores than Swiss controls for physical functioning (48.2 vs. 53.1) and general health perception (51.2 vs. 56.5). Patients with other disorders had lower physical functioning scores (46.9 vs. 53.1) and PCS scores (50.2 vs. 54.9) than Swiss controls.

**Table 3:** SF-36 mean T-scores and confidence intervals of rhGH treated patients stratified by indications and compared with Swiss controls.

		Group I		Group II				Group III	Swiss controls	
		IGHD (n = 112)	ISS (n = 17)	Turner (n = 47)	MPHD (n = 40)	SGA (n = 18)	Other^a^ (n = 23)	Cancer (n = 43)	(n = 695)	p-value^b^
*Physical functioning*	Mean	52.0	54.0	51.0	48.2	50.4	46.9	36.0	53.1	<0.001
	95% CI	50.9, 53.1	52.8, 55.1	49.2, 52.8	43.4, 53.0	46.9, 53.9	41.9, 52.0	31.0, 41.0	52.6, 53.6	
*Bodily pain*	Mean	55.2	59.7	59.0	55.8	58.0	53.2	53.3	56.5	<0.001
	95% CI	53.5, 56.8	57.2, 62.2	57.1, 61.0	53.2, 58.4	56.1, 59.9	49.3, 57.1	50.6, 56.1	55.8, 57.2	
*Role limitation physical*	Mean	50.6	53.3	49.7	49.5	49.9	50.2	43.1	50.9	<0.001
	95% CI	49.6, 51.6	51.8, 54.8	47.3, 52.1	47.0, 52.1	47.0, 52.9	47.0, 53.4	40.1, 46.0	50.3, 51.5	
*Energy & vitality*	Mean	53.3	61.3	56.2	53.8	55.8	56.6	50.9	55.3	0.163
	95% CI	50.6, 56.0	54.2, 68.4	53.1, 59.3	50.0, 57.5	49.5, 62.2	56.6, 62.6	47.2, 54.6	53.9, 56.6	
*Mental health*	Mean	52.1	55.7	53.2	51.1	55.0	53.0	51.0	54.2	0.266
	95% CI	49.8, 54.5	50.1, 61.3	50.5, 55.9	48.4, 53.8	48.7, 61.2	46.8, 59.2	47.7, 54.2	53.1, 55.3	
*General health perception*	Mean	53.6	61.5	53.5	51.2	54.5	52.4	46.7	56.5	<0.001
	95% CI	51.6, 55.6	57.3, 65.7	50.2, 56.8	47.2, 55.3	50.5, 58.5	48.0, 56.8	42.3, 51.0	55.5, 57.5	
*Role limitation emotional*	Mean	49.8	52.6	48.8	47.7	47.2	48.2	46.9	48.7	<0.001
	95% CI	48.4, 51.1	51.3, 53.9	46.4, 51.3	45.0, 50.4	42.6, 51.9	44.2, 52.2	44.2, 49.6	47.8, 49.6	
*Social functioning*	Mean	50.6	53.0	52.9	51.0	49.4	49.3	44.3	50.8	0.013
	95% CI	48.8, 52.4	49.1, 56.9	50.5, 55.3	48.6, 53.5	45.1, 53.7	44.6, 54.1	40.8, 47.8	49.9, 51.6	
*PCS*	Mean	53.2	57.4	53.8	52.1	53.4	50.2	42.6	54.9	<0.001
	95% CI	52.0, 54.4	55.5, 59.2	51.6, 55.9	48.5, 55.7	51.6, 55.2	46.7, 53.8	39.0, 46.2	54.3, 55.5	
*MCS*	Mean	50.9	54.9	52.3	50.2	51.5	52.3	51.3	51.3	0.872
	95% CI	48.6, 53.2	49.6, 60.2	49.5, 55.2	47.5, 52.9	45.5, 57.5	46.3, 58.4	48.1, 54.5	50.1, 52.5	

Higher T-scores indicate higher HRQoL (expected mean from German norm population = 50, SD = 10).

Abbreviations: 95% CI, 95% confidence interval; IGHD, isolated growth hormone deficiency; ISS, idiopathic short stature; MCS, mental component summary; MPHD, multiple pituitary hormone deficiency; n, number; PCS, physical component summary; rhGH, recombinant human growth hormone; SF-36, Short Form-36; SGA, small for gestational age.

^a^Other disorders include: clinically defined syndromes (except Turner syndrome), skeletal dysplasia, disorders in organ systems, osteogenesis imperfecta, central diabetes insipidus, congenital adrenal hyperplasia.

^b^Global p-values were calculated from linear regression models, testing if the variable ‘indication group’ as a whole was associated with SF-36 mean T-scores

### Factors associated with the Physical Component Summary (PCS)

In the univariable linear regression, a high educational level was associated with higher PCS. But patients <20 years at time of study, patients from Group III, or patients who had received an rhGH dose <30 μg/kg/day scored lower in the PCS. We found no association for sex, age at start or end of rhGH treatment, treatment duration, height gain, and final height (Table 4). In the multivariable linear regression, reaching a tertiary education was associated with a higher score in the PCS, while being in Group III was associated with a lower PCS. Sex, age, rhGH dose, treatment duration, height gain, and final height were not significantly associated with the PCS (Table 4).

**Table 4:** Factors associated with the SF-36 Physical Component Summary in rhGH treated patients (n = 300).

	Univariable regression			Multivariable regression		
	coeff	95% CI	p-value^a^	coeff	95% CI	p-value^a^
*Sex*			0.221			0.604
Male	ref			ref		
Female	-1.31	-3.41, 0.79		-0.58	-2.86, 1.70	
*Current age (years)*			0.002			0.969
<20	-3.68	-6.09, -1.26		0.06	-2.75, 2.87	
20–25	ref			ref		
>25	0.81	-1.73, 3.35		0.34	-2.49, 3.18	
*Education*			0.001			0.018
Primary	-0.63	-3.02, 1.76		-1.86	-4.71, 0.99	
Secondary/Unknown	ref			ref		
Tertiary	4.78	2.12, 7.42		2.84	0.002, 5.68	
*Indication group* ^b^			<0.001			<0.001
Group I	ref			ref		
Group II	-1.24	-3.30, 0.81		-0.10	-2.40, 2.20	
Group III	-11.17	-14.13, -8.21		-20.09	-26.59, -13.59	
*rhGH dose (μg/kg/day)*			<0.001			0.548
<30	-4.94	-7.43, -2.44		-1.60	-4.69, 1.50	
30–50	ref			ref		
>50	-0.19	-3.62, 3.25		-0.78	-4.08, 2.53	
*Age at start of treatment*	0.12	-0.19, 0.44	0.445	n.a.	n.a.	n.a.
*Age at end of treatment*	-0.23	-0.61, 0.15	0.241	n.a.	n.a.	n.a.
*Treatment duration*	-0.18	-0.46, 0.09	0.191	-0.06	-0.42, 0.29	0.719
*Height gain*	1.05	-0.02, 2.13	0.054	0.10	-1.35, 1.55	0.888
*Final height*	0.96	-0.16, 2.08	0.092	0.43	-0.91, 1.78	0.506

Abbreviations: 95% CI, 95% confidence interval; GHD, growth hormone deficiency; IGHD, isolated growth hormone deficiency; ISS, idiopathic short stature; n, number; n.a., not applicable / not available; ref, reference; rhGH, recombinant human growth hormone; SF-36, Short Form-36.

^a^Global p-values calculated with likelihood ratio test.

^b^Group I includes healthy patients with IGHD or ISS; Group II patients with associated diseases or syndromes; Group III childhood cancer survivors with GHD.

### Factors associated with the Mental Component Summary (MCS)

None of these factors was associated with the MCS in the univariable linear regression (S3 Table). In the multivariable linear regression, patients who were <20 years years at time of study scored higher in the MCS. Sex, education, indication group, rhGH dose, treatment duration, height gain and final height were not significantly associated (S3 Table).

## Discussion

This large, representative study found that HRQoL depended mainly on the underlying indication for rhGH treatment. In patients with IGHD or ISS, HRQoL was similar to Swiss controls. In patients with associated diseases or syndromes, it was slightly lower, mostly in the physical health scales: those treated with rhGH had a PCS of 52.5, while Swiss controls had a PCS of 54.9. Cancer patients had the lowest scores in all scales. Their results for four out of eight scales (physical functioning, role limitation physical, general health perception and social functioning) and the PCS were significantly lower than for Swiss controls. PCS was as low as 42.6. We found no association between final height and HRQoL.

### Findings of other studies on health-related quality of life in young adults with childhood onset rhGH treatment

When we compared the whole group of rhGH treated patients to Swiss controls, we found that the HRQoL of patients was impaired mainly in the physical scales of the SF-36. Sandberg et al. [6] and Lagrou et al. [5] had similar results.

When we looked at single indications only, we found similar results to Sandberg et al. for healthy patients with IGHD [6]. These patients had a comparable HRQoL to Swiss controls in all scales, except of a lower general health perception and PCS. Our findings for patients with ISS, who had a similar or better HRQoL than Swiss controls, are comparable to those of a study by Rekers-Mombark et al. [19], which found that the HRQoL of young adults with ISS treated with rhGH was similar to the HRQoL of untreated adults with ISS, and to the normal population. For women with Turner syndrome, we found that HRQoL was comparable to Swiss controls, except for lower physical functioning and less bodily pain. Also, a French study by Carel et al. found a normal HRQoL among Turner women [21], and a Canadian study found no differences between treated and untreated Turner patients [20]. A Swedish study reported that Turner women were more socially isolated, but in less pain than randomly selected, age-matched women [24]. Dutch researchers reported that women with Turner syndrome had higher HRQoL than the general population in social functioning, role emotional and bodily pain [22]. For patients with MPHD, HRQoL was lower than in Swiss controls for physical functioning and general health perception. Other studies compared HRQoL between MPHD and IGHD patients, with variable findings: some found lower HRQoL in MPHD patients [5, 6]; others did not [25].

Discrepancies between our findings and those of other studies may have been caused by differences in the populations under study, the comparison groups, or the instruments to assess HRQoL in GH deficient children or adults [26]. This is a general problem for studies on long-term outcomes of rhGH treatment, and can only be solved by conducting further cohort studies in a standardized way.

It was striking that survivors of childhood cancer (Group III) had the lowest HRQoL of all rhGH treated patients and Swiss controls. Most (65%) of those patients had been diagnosed with a CNS tumour (S1 Table). In a recent post-marketing study, adult patients with childhood-onset craniopharyngeoma had HRQoL similar to patients with childhood-onset extrasellar tumours, or to patients with childhood-onset idiopathic congenital hypopituitarism [27]. But a previous analysis from the same database found that adult patients with craniopharyngeoma had lower HRQoL than the norm population [28]. Patients with central nervous system tumours are likely to develop chronic health problems [29] that, in turn, may affect HRQoL. Our group recently conducted a study of childhood cancer survivors and identified a range of chronic health problems that negatively influenced HRQoL [12]. Endocrinopathies, neurologic complications, or other late effects reported by those patients may decrease their HRQoL [30].

### Limitations and strengths of the study

A limitation we share with most other studies on HRQoL in GHD is the lack of an untreated control group of patients with the same diagnoses, since it is unethical to withhold necessary treatment with rhGH from patients who require it. Sandberg et al. [6] compared patients to their untreated siblings, but we did not have this option. Normal populations may also serve as a comparison group, but may differ from the investigated population by year of assessment, socio-demographic factors or cultural background [31]. Since Swiss norm data was not available, we used Germany norm data [14]. We also compared rhGH treated patients with Swiss healthy untreated siblings of childhood cancer patients. However, all studies that use siblings of sick patients as control groups suffer limitations, since those siblings, because they lived with sick patients, may have lower HRQoL than their peers in the general population. On the other hand, it is also possible that siblings reported a higher HRQoL, since they compared their own HRQoL to that of their sick siblings [32].

Our response rate was only 58% (rhGH patients) and 51% (Swiss controls), despite sending a postal reminder to both groups and reminding rhGH patients by phone. Non-participants were similar to participants in their socio-demographic and treatment characteristics, but we cannot rule out self-selection bias among both rhGH treated participants and the Swiss controls.

Our study was strengthened by its inclusion of all types of patients treated with rhGH in childhood. This enabled us to compare HRQoL among patients with different indications. We also included patients treated both in university hospitals and in private practice, so our dataset represented the entire range of rhGH patients treated in Switzerland. While most other studies on childhood rhGH treatment assessed short-term HRQoL during the patients’ growing period, our study investigated long-term results after patients reached their adult height.

### Interpretation of results and implication for practice

We identified patients with associated disorders and cancer survivors as subgroups at risk for low HRQoL. Those patients may benefit from follow-up and psychological counselling. We also found that the education of rhGH treated participants is a determinant of HRQoL, with higher educated participants having a better quality of life. This association is well known and reported previously [33]. We thought it remarkable that final height was not associated with HRQoL, since HRQoL is generally believed to be lower in shorter persons [34]. We, however, found no evidence that this assumption is true. A large longitudinal population-based cohort study on the health of children and adolescents in Germany found that height had negligible influence on HRQoL [35]. The same was demonstrated for adults in a nationwide population study from France [36]. To determine whether GHD or the resulting short stature is responsible for reducing HRQoL, Stabler compared the incidence of social phobia in formerly treated GHD patients, short non-treated sex- and age-matched adults, and the normal population [37]. He found that GHD patients had a higher incidence of social phobia, and short non-GHD people had a lower incidence of social phobia than the general population. Thus, reduced HRQoL may be related to GHD indication, rather than to low final height.

## Conclusion

We suggest that future research takes into account the influence of the underlying indication when interpreting results, since our findings indicate that HRQoL after treatment with rhGH in childhood is mainly determined by its underlying indication. HRQoL was normal in patients with IGHD or ISS, slightly reduced in patients with associated diseases or syndromes, and clearly lower in former childhood cancer patients than in Swiss controls.

## Supporting Information

S1 Table Underlying indications for rhGH treatment in the study population. (PDF)

S2 Table SF-36 mean T-scores and confidence intervals of rhGH treated patients compared with Swiss controls. (PDF)

S3 Table Factors associated with the SF-36 Mental Component Summary in rhGH treated patients (n = 300). (PDF)

## References

[1] Molitch ME, Clemmons DR, Malozowski S, Merriam GR, Vance ML, Endocrine S. Evaluation and treatment of adult growth hormone deficiency: an Endocrine Society clinical practice guideline. The Journal of clinical endocrinology and metabolism. 2011;96(6):1587–1609. 10.1210/jc.2011-0179 21602453

[2] Hazem A, Elamin MB, Bancos I, Malaga G, Prutsky G, Domecq JP, et al Body composition and quality of life in adults treated with GH therapy: a systematic review and meta-analysis. European journal of endocrinology / European Federation of Endocrine Societies. 2012;166(1):13–20. 10.1530/EJE-11-0558 21865409

[3] Hoybye C, Christiansen JS. Growth hormone replacement in adults—current standards and new perspectives. Best practice & research Clinical endocrinology & metabolism. 2015;29(1):115–123. 25617177 10.1016/j.beem.2014.09.006

[4] Bullinger M, Koltowska-Haggstrom M, Sandberg D, Chaplin J, Wollmann H, Noeker M, et al Health-related quality of life of children and adolescents with growth hormone deficiency or idiopathic short stature—part 2: available results and future directions. Hormone research. 2009;72(2):74–81. 10.1159/000232159 19690424

[5] Lagrou K, Xhrouet-Heinrichs D, Massa G, Vandeweghe M, Bourguignon JP, De Schepper J, et al Quality of life and retrospective perception of the effect of growth hormone treatment in adult patients with childhood growth hormone deficiency. Journal of pediatric endocrinology & metabolism: JPEM. 2001;14 Suppl 5:1249–1260; discussion 1261–1242. 11964020

[6] Sandberg DE, MacGillivray MH, Clopper RR, Fung C, LeRoux L, Alliger DE. Quality of life among formerly treated childhood-onset growth hormone-deficient adults: a comparison with unaffected siblings. The Journal of clinical endocrinology and metabolism. 1998;83(4):1134–1142. 9543130 10.1210/jcem.83.4.4712

[7] Visser-van Balen H, Geenen R, Looij J, Huisman J, Wit JM, Sinnema G. The views of young adults and their parents on hormone treatment for short stature in adolescence. Hormone research. 2008;69(3):172–179. 10.1159/000112591 18219221

[8] Wiren L, Johannsson G, Bengtsson BA. A prospective investigation of quality of life and psychological well-being after the discontinuation of GH treatment in adolescent patients who had GH deficiency during childhood. The Journal of clinical endocrinology and metabolism. 2001;86(8):3494–3498. 11502769 10.1210/jcem.86.8.7709

[9] Vahl N, Juul A, Jorgensen JO, Orskov H, Skakkebaek NE, Christiansen JS. Continuation of growth hormone (GH) replacement in GH-deficient patients during transition from childhood to adulthood: a two-year placebo-controlled study. The Journal of clinical endocrinology and metabolism. 2000;85(5):1874–1881. 10843168 10.1210/jcem.85.5.6598

[10] Stouthart PJ, Deijen JB, Roffel M, Delemarre-van de Waal HA. Quality of life of growth hormone (GH) deficient young adults during discontinuation and restart of GH therapy. Psychoneuroendocrinology. 2003;28(5):612–626. 12727130 10.1016/s0306-4530(02)00045-8

[11] Kuehni CE, Rueegg CS, Michel G, Rebholz CE, Strippoli MP, Niggli FK, et al Cohort profile: the Swiss childhood cancer survivor study. International journal of epidemiology. 2012;41(6):1553–1564. 10.1093/ije/dyr142 22736394

[12] Rueegg CS, Gianinazzi ME, Rischewski J, Beck Popovic M, von der Weid NX, Michel G, et al Health-related quality of life in survivors of childhood cancer: the role of chronic health problems. Journal of cancer survivorship: research and practice. 2013;7(4):511–522. 23784593 10.1007/s11764-013-0288-4

[13] Wengenroth L, Rueegg CS, Michel G, Essig S, Ammann RA, Bergstraesser E, et al Life partnerships in childhood cancer survivors, their siblings, and the general population. Pediatric blood & cancer. 2014;61(3):538–545. 24136901 10.1002/pbc.24821 PMC5917072

[14] Ellert U, Bellach BM. [The SF-36 in the Federal Health Survey—description of a current normal sample]. Gesundheitswesen. 1999;61 Spec No:S184–190. 10726419

[15] Wit JM, Ranke MB, Kelnar CJH. The ESPE classification of paediatric endocrine diagnoses—Foreword. Hormone research. 2007;68:Vii-+.

[16] Steliarova-Foucher E, Stiller C, Lacour B, Kaatsch P. International Classification of Childhood Cancer, third edition. Cancer. 2005;103(7):1457–1467. 15712273 10.1002/cncr.20910

[17] Kuehni CE, Strippoli MP, Rueegg CS, Rebholz CE, Bergstraesser E, Grotzer M, et al Educational achievement in Swiss childhood cancer survivors compared with the general population. Cancer. 2012;118(5):1439–1449. 10.1002/cncr.26418 21823113

[18] Ware JE Jr, Gandek B. Overview of the SF-36 Health Survey and the International Quality of Life Assessment (IQOLA) Project. Journal of clinical epidemiology. 1998;51(11):903–912. 9817107 10.1016/s0895-4356(98)00081-x

[19] Rekers-Mombarg LT, Busschbach JJ, Massa GG, Dicke J, Wit JM. Quality of life of young adults with idiopathic short stature: effect of growth hormone treatment. Dutch Growth Hormone Working Group. Acta paediatrica (Oslo, Norway: 1992). 1998;87(8):865–870. 10.1080/080352598750013653 9736235

[20] Taback SP, Van Vliet G. Health-related quality of life of young adults with Turner syndrome following a long-term randomized controlled trial of recombinant human growth hormone. BMC pediatrics. 2011;11:49 10.1186/1471-2431-11-49 21619701 PMC3125334

[21] Carel JC, Ecosse E, Bastie-Sigeac I, Cabrol S, Tauber M, Leger J, et al Quality of life determinants in young women with turner's syndrome after growth hormone treatment: results of the StaTur population-based cohort study. The Journal of clinical endocrinology and metabolism. 2005;90(4):1992–1997. 15644402 10.1210/jc.2004-1395

[22] Bannink EM, Raat H, Mulder PG, de Muinck Keizer-Schrama SM. Quality of life after growth hormone therapy and induced puberty in women with Turner syndrome. The Journal of pediatrics. 2006;148(1):95–101. 16423606 10.1016/j.jpeds.2005.08.043

[23] Robinson LD, Jewell NP. Covariate adjustment. Biometrics. 1991;47(1):342–343. 2049511

[24] Amundson E, Boman UW, Barrenas ML, Bryman I, Landin-Wilhelmsen K. Impact of growth hormone therapy on quality of life in adults with turner syndrome. The Journal of clinical endocrinology and metabolism. 2010;95(3):1355–1359. 10.1210/jc.2009-1754 20080847

[25] Attanasio AF, Shavrikova EP, Blum WF, Shalet SM. Quality of life in childhood onset growth hormone-deficient patients in the transition phase from childhood to adulthood. The Journal of clinical endocrinology and metabolism. 2005;90(8):4525–4529. 15899956 10.1210/jc.2005-0439

[26] Brutt AL, Sandberg DE, Chaplin J, Wollmann H, Noeker M, Koltowska-Haggstrom M, et al Assessment of health-related quality of life and patient satisfaction in children and adolescents with growth hormone deficiency or idiopathic short stature—part 1: a critical evaluation of available tools. Hormone research. 2009;72(2):65–73. 10.1159/000232158 19690423

[27] Yuen KC, Koltowska-Haggstrom M, Cook DM, Fox JL, Jonsson PJ, Geffner ME, et al Clinical characteristics and effects of GH replacement therapy in adults with childhood-onset craniopharyngioma compared with those in adults with other causes of childhood-onset hypothalamic-pituitary dysfunction. European journal of endocrinology / European Federation of Endocrine Societies. 2013;169(4):511–519. 10.1530/EJE-13-0280 23904277

[28] Kendall-Taylor P, Jonsson PJ, Abs R, Erfurth EM, Koltowska-Haggstrom M, Price DA, et al The clinical, metabolic and endocrine features and the quality of life in adults with childhood-onset craniopharyngioma compared with adult-onset craniopharyngioma. European journal of endocrinology / European Federation of Endocrine Societies. 2005;152(4):557–567. 15817911 10.1530/eje.1.01877

[29] Oeffinger KC, Mertens AC, Sklar CA, Kawashima T, Hudson MM, Meadows AT, et al Chronic health conditions in adult survivors of childhood cancer. The New England journal of medicine. 2006;355(15):1572–1582. 17035650 10.1056/NEJMsa060185

[30] Armstrong GT. Long-term survivors of childhood central nervous system malignancies: the experience of the Childhood Cancer Survivor Study. European journal of paediatric neurology: EJPN: official journal of the European Paediatric Neurology Society. 2010;14(4):298–303. 20110182 10.1016/j.ejpn.2009.12.006 PMC2885448

[31] Bowling A, Bond M, Jenkinson C, Lamping DL. Short Form 36 (SF-36) Health Survey questionnaire: which normative data should be used? Comparisons between the norms provided by the Omnibus Survey in Britain, the Health Survey for England and the Oxford Healthy Life Survey. Journal of public health medicine. 1999;21(3):255–270. 10528952 10.1093/pubmed/21.3.255

[32] Strack F, Schwarz N, Chassein B, Kern D, Wagner D. Salience of comparison standards and the activation of social norms: consequences for judgments of happiness and their communication. British Journal of Social Psychology. 1990;29(4):303–314.

[33] Furnee CA, Groot W, van den Brink HM. The health effects of education: a meta-analysis. European journal of public health. 2008;18(4):417–421. 10.1093/eurpub/ckn028 18434381

[34] Christensen TL, Djurhuus CB, Clayton P, Christiansen JS. An evaluation of the relationship between adult height and health-related quality of life in the general UK population. Clinical endocrinology. 2007;67(3):407–412. 17573903 10.1111/j.1365-2265.2007.02901.x

[35] Sommer R, Daubmann A, Quitmann J, Ravens-Sieberer U, Bullinger M. Understanding the impact of statural height on health-related quality of life in German adolescents: a population-based analysis. European journal of pediatrics. 2014 10.1007/s00431-014-2480-6 25535173

[36] Coste J, Pouchot J, Carel JC. Height and health-related quality of life: a nationwide population study. The Journal of clinical endocrinology and metabolism. 2012;97(9):3231–3239. 10.1210/jc.2012-1543 22745240

[37] Stabler B. Impact of growth hormone (GH) therapy on quality of life along the lifespan of GH-treated patients. Hormone research. 2001;56 Suppl 1:55–58. 11786687 10.1159/000048136

