# Supplementary material for: Health-Related Quality of Life of Young Adults Treated with Recombinant Human Growth Hormone during Childhood
Source: PLoS One. 2015 Oct 16;10(10):e0140944. doi: 10.1371/journal.pone.0140944 (PMC4608786; doi:10.1371/journal.pone.0140944)
Supplement: S1 Table — (PDF) [file pone.0140944.s001.pdf]

S1 Table. Underlying indications for rhGH treatment in the study population.

Indication group	Underlying indication for rhGH treatment[a]	n
Group I	Isolated growth hormone deficiency	112
	Idiopathic short stature	17
Group II	Turner syndrome	47
	Multiple pituitary hormone deficiency	40
	Short for gestational age	18
	Other	
	-Clinically defined syndromes (except Turner syndrome)	11
	-Skeletal dysplasia	6
	-Disorders in organ systems	2
	-Osteogenesis imperfecta	2
	-Central diabetes insipidus	1
	-Congenital adrenal hyperplasia	1
Group III	Bone tumour	2
	Central nervous system tumour	28
	Germ cell tumour	3
	Leukaemia	2
	Neuroblastoma	2
	Soft tissue sarcoma	6

Abbreviations: n, number; rhGH, recombinant human growth hormone

[a]Indications of Groups I and II were classified using the coding system of the European Society for Pediatric Endocrinology (ESPE). For Group III, the International Classification of Childhood Cancer (ICCC-3) was used
