# Supplementary material for: Health-Related Quality of Life of Young Adults Treated with Recombinant Human Growth Hormone during Childhood
Source: PLoS One. 2015 Oct 16;10(10):e0140944. doi: 10.1371/journal.pone.0140944 (PMC4608786; doi:10.1371/journal.pone.0140944)
Supplement: S2 Table — (PDF) [file pone.0140944.s002.pdf]

**S2 Table. SF-36 mean T-scores and confidence intervals of rhGH treated patients compared with Swiss controls.**

| | | rhGH patients (n=300) | Swiss controls (n=695) | p-value[a] |
|---|---|---|---|---|
| *Physical functioning* | Mean | 48.7 | 53.1 | <0.001 |
| | 95% CI | 47.4, 50.1 | 52.6, 53.6 | |
| *Bodily pain* | Mean | 55.9 | 56.5 | 0.296 |
| | 95% CI | 54.9, 56.8 | 55.8, 57.2 | |
| *Role limitation physical* | Mean | 49.3 | 50.9 | 0.004 |
| | 95% CI | 48.5, 50.2 | 50.3, 51.5 | |
| *Energy & vitality* | Mean | 54.3 | 55.3 | 0.386 |
| | 95% CI | 52.8, 55.9 | 53.9, 56.6 | |
| *Mental health* | Mean | 52.5 | 54.2 | 0.054 |
| | 95% CI | 51.1, 53.8 | 53.1, 55.3 | |
| *General health perception* | Mean | 52.8 | 56.5 | <0.001 |
| | 95% CI | 51.4, 54.1 | 55.5, 57.5 | |
| *Role limitation emotional* | Mean | 48.8 | 48.7 | 0.815 |
| | 95% CI | 47.9, 49.8 | 47.8, 49.6 | |
| *Social functioning* | Mean | 50.1 | 50.8 | 0.375 |
| | 95% CI | 49.0, 51.2 | 49.9, 51.6 | |
| *PCS* | Mean | 51.7 | 54.9 | <0.001 |
| | 95% CI | 50.7, 52.8 | 54.3, 55.5 | |
| *MCS* | Mean | 51.5 | 51.3 | 0.873 |
| | 95% CI | 50.2, 52.8 | 50.1, 52.5 | |

Higher T-scores indicate higher HRQoL (expected mean from German norm population=50, SD=10).

Abbreviations: 95% CI, 95% confidence interval; MCS, mental component summary; n, number; PCS, physical component summary; rhGH, recombinant human growth hormone; SF-36, Short Form-36.
[a]p-values calculated from linear regression models.
