# Supplementary material for: Health-Related Quality of Life of Young Adults Treated with Recombinant Human Growth Hormone during Childhood
Source: PLoS One. 2015 Oct 16;10(10):e0140944. doi: 10.1371/journal.pone.0140944 (PMC4608786; doi:10.1371/journal.pone.0140944)
Supplement: S3 Table — (PDF) [file pone.0140944.s003.pdf]

S3 Table. Factors associated with the SF-36 Mental Component Summary in rhGH treated patients (n=300).

	Univariable regression			Multivariable regression		
	**coeff**	**95% CI**	**p-value[a]**	**coeff**	**95% CI**	**p-value[a]**
Sex			0.738			0.845
Male	ref			ref		
Female	-0.46	-3.15, 2.24		0.33	-3.16, 3.83	
Current age (years)			0.102			0.021
<20	3.33	0.20, 6.46		5.68	1.37, 9.99	
20-25	ref			ref		
>25	0.78	-2.52, 4.07		0.35	-4.00, 4.69	
Education			0.956			0.251
Primary	-0.47	-3.61, 2.67		-3.53	-7.90, 0.85	
Secondary/Unknown	ref			ref		
Tertiary	-0.27	-3.74, 3.21		-1.50	-5.86, 2.86	
Indication group[b]			0.989			0.822
Group I	ref			ref		
Group II	0.15	-2.73, 3.03		-1.03	-4.57, 2.50	
Group III	-1.34	-4.28, 4.01		0.26	-9.70, 10.23	
rhGH dose (µg/kg/day)			0.431			0.520
<30	0.15	-3.03, 3.33		-2.11	-6.85, 2.63	
30-50	ref			ref		
>50	-2.76	-7.14, 1.62		-2.09	-7.26, 2.97	
Age at start of treatment	-0.09	-0.50, 0.31	0.651	n.a.		n.a.
Age at end of treatment	0.06	-0.43, 0.54	0.824	n.a.		n.a.
Treatment duration	0.10	-0.25, 0.45	0.570	-0.15	-0.70, 0.39	0.563
Height gain	0.04	-1.30, 1.39	0.949	0.75	-1.47, 2.97	0.489
Final height	-0.22	-1.76, 1.33	0.782	-0.69	-2.75, 1.47	0.495

Abbreviations: 95% CI, 95% confidence interval; GHD, growth hormone deficiency; IGHD, isolated growth hormone deficiency; ISS, idiopathic short stature; n, number; n.a., not applicable / not available; ref, reference; rhGH, recombinant human growth hormone; SF-36, Short Form-36.

[a]Global p-values calculated with likelihood ratio test.

[b]Group I includes healthy patients with IGHD or ISS; Group II patients with associated diseases or syndromes; Group III childhood cancer survivors with GHD.
